# Depression by gender and associated factors among older adults in India: implications for age-friendly policies

**DOI:** 10.1038/s41598-023-44762-8

**Published:** 2023-10-17

**Authors:** Ronak Paul, T. Muhammad, Rashmi Rashmi, Palak Sharma, Shobhit Srivastava, Preeti Pushpalata Zanwar

**Affiliations:** 1 International Institute for Population Sciences, Mumbai, 400088 India; 2 Thomas Jefferson University, Philadelphia, PA USA

**Keywords:** Geriatrics, Depression

## Abstract

Inspite of implementing policies to control mental health problems, depression remains a severe health concern among older adults in India. We examined self-reported differences in the depression among older men and women in India and examined associated factors for gender differences in depression at the population level. We utilized nationally representative data from the Longitudinal Aging Study in India (LASI) wave I, for years 2017–2019. Our analytical sample comprised of 30,637 older adults ages 60 years and above (14,682 men and 15,655 women). We conducted descriptive statistics and Chi-Square tests followed by binary logistic regression and multivariate decomposition analyses to examine our study objectives. Depression was reported in − 7.4% (95% CI 7.0, 7.8) of older men and 9.5% (CI 9.1, 10.0) of older women. Poor self-rated health, multimorbidity status, physical activity, difficulty in activities of daily living (ADL) and instrumental ADL (IADL) were the significant health-related factors associated with depression among older men and women. Not being satisfied with one’s life, not being satisfied with their present living arrangement, receiving any type of ill-treatment, and being widowed were the significant factors associated with depression among older men and women. We found gender disparity in self-reported depression. Marital status contributed-to 36.7% of the gender gap in depression among older adults. Additionally, ADL and IADL difficulties among men and women contributed to 17.6% and 34.0%, gender gap, self-rated health contributed to 18.8% gap, whereas not having equal social participation (4.4%) and not satisfied in present living arrangements (8.1%) were other factors that contributed to gender gap for depression in India. Depression is a critical and persistent public health problem among—older females in India. Our findings provide a broader framework for policymakers and health practitioners to focus on gender-specific strategies to mitigate this highly emergent problem.

Depression is the most prevalent psychiatric disorder globally and is an emerging public health concern. Despite prioritizing policies and programs to control mental health problems, depression is a common illness affecting an estimated 280 million people globally^1^. In 2011, the World Mental Health Survey from 17 countries found -one in 20 people experienced an episode of depression^2^. Depression can cause tremendous suffering to an individual leading to disability and death. Globally, approximately 700,000 annual suicide deaths are attributed to depression^1,2^.

The recent rise in depression prevalence in low-and middle-income countries (LMICs) can be explained by the rapid aging of the population and multiple risks of everyday life stressors^1,3–6^. Ample evidence shows -depression is more prevalent among females than males in India^7–11^. India is embedded in gender stereotypes, with preferential treatment for boys culturally and throughout life course at every level, domain, and sector. These preferences, can largely explain the increased prevalence of depressive symptoms among women^12–14^.

Although the higher prevalence of depressive symptoms among women is associated with gender-related socio-cultural norms such as chastity, masculinity and caste beliefs in the country^7,15–17^, the contextual factors also contribute to the female disadvantage in mental health that include limited educational opportunities and poor socioeconomic background^18–20^. Nevertheless, gender brings a curse to men too. For instance, research in India showed that the association of multiple chronic conditions with depressive symptoms is more pronounced in older men than women^21,22^. Similarly, a study among older adults in Korea showed that household wealth, unlike income and education, was significantly associated with higher depressive symptoms in men but not in women^23^. Additionally, social isolation, living alone and lifestyle behaviours, such as smoking, drinking alcohol and physical inactivity are highly associated with the risk of depression among older men^24,25^.

Although being female is a crucial risk factor for depression, there is growing evidence for lifestyle diseases and complex social, psychological, and biological factors triggered by major life events like retirement, bereavement, stress, and psychological trauma have been reported to increase the risk of depressive symptoms for both men and women in India^3–6^. Differential impacts of these factors highlight the need to understand the contributing factors for differences in population level depression patterns among older men and women in India. We therefore aimed to examine the factors associated with differences in self-reported depression -among older men and women in India.

## Methods

### Data source

We utilized data from the longitudinal aging study in India (LASI), wave-I -from 2017 to 2019. The LASI is the sister study of the United States Health and Retirement Studies (HRS). It is a nationally representative survey conducted by the International Institute for Population Sciences (IIPS) in collaboration with the Harvard T.H. Chan School of Public Health and the University of Southern California (USC). LASI provides vital information on demography, chronic health conditions, symptom-based health conditions, functional health, mental health (cognition and depression), household economic status, healthcare utilization and health insurance, family and social networks, work and employment, retirement and life expectations on 72,250 older adults ages 45 and above across all the States and Union Territories of India. LASI has a multistage stratified cluster sampling design and plans to follow the sample biennially over the next 25 years. The survey uses a face-to-face Computer-Assisted Personal Interview (CAPI) for respondent interviews and data collection. The individual response rates of the survey ranges from 96% in Nagaland to 74% in Chandigarh. Further details regarding the sample design, survey instruments, fieldwork, data collection and processing, informed consent and response rates are publicly available in the LASI report and the following website: https://www.iipsindia.ac.in/sites/default/files/LASI_India_Report_2020_compressed.pdf^26^.

Our study is based on a sample of 31,464 older adults (15,098 men and 16,366 women) in India aged 60 and over. We omitted 827 older adults on whom data on self-reported depression was not available. This reduced our final = analytical sample—to 30,637 older adults, -of which 14,682 -were men and 15,655 were women.

### Ethics statement

We utilized publicly available data with de-identified respondents. The Indian Council of Medical Research (ICMR) extended the necessary guidance and ethical approval for conducting the LASI. During the survey, LASI interviewers obtained informed consent from the respondents before data collection. The survey was implemented following the relevant guidelines and regulations.

### Outcome variable(s)

Our primary binary outcome variable was the presence of major depression among older adults. Major depressive disorder was assessed using the Composite International Diagnostic Interview short-form (CIDI-SF) scale, widely used to diagnose psychiatric depression^26,27^. The LASI CIDI-SF questionnaire included the following ten questions:

- During the last 12 months, was there ever a time when you felt sad, blue, or depressed for two weeks or more in a row?
- Please think of the two weeks during the last 12 months when these feelings were worst. During that time, did the feelings of being sad, blue, or depressed usually last all day long, most of the day, about half the day, or less than half the day?
- During those 2 weeks, did you feel this way every day, almost every day, or less often than that?
- During those 2 weeks, did you lose interest in most things?
- During those 2 weeks, did you ever feel more tired out or low in energy than is usual?
- During those 2 weeks, did you lose your appetite?
- During those 2 weeks, did you have a lot more trouble concentrating than usual?
- During those two weeks, did you feel down on yourself and worthless?
- During those 2 weeks, did you think about your death or someone else’s in general?
- During those 2 weeks, did you have more trouble falling asleep than usual?

The response for all the items (except items “b” and “c”) was binary, i.e., in “No” (coded as 0) and “Yes” (coded as 1). Individuals who felt sad, blue or depressed “all day long” or “most of the day” were coded as “Yes”; else, they were coded as “No.” Similarly, individuals who felt sad, blue or depressed “every day” or “almost every day” were coded as “Yes”; else, they were coded as “No”. The ten items had a Cronbach’s α reliability coefficient of 0.70. The ten items were summed to obtain the depression scale, with scores ranging from 0 to 10. We classified older adults with a 5+ score—as “Depressed” and those with a score of 4 and below as “Not depressed”^28–30^.

### Group variable

The proportion of older adults who self-report depression may differ in men vs. women in India due to differences in socioeconomic, demographic and morbidity characteristics. Notably, LASI collected information from respondents regarding their gender, which was recorded as “male” and “female.” In our study, we refer to “male” and “female” as “men” and “women”.

### Independent variables

Based on prior research, we included health-related^30–32^, socio-demographic and household characteristics^33–35^ as factors that could explain the presence of major depression among older adults in India. The individual health-related characteristics included were: -

- Self-rated health -categorized as good, average, or poor^36^.
- Chronic morbidity status—categorized as no condition, single condition, or multiple conditions. LASI collected information on whether an older adult was ever diagnosed with hypertension or high blood pressure, diabetes or high blood pressure, cancer or malignant tumour, chronic lung diseases, chronic heart diseases, stroke, bone or joint diseases, neurological or psychological problems, and high cholesterol. Individuals having no diseases, any one of the diseases and two or more diseases were categorized into “no condition,” “single condition,” and “multiple conditions”^37^.
- Physical activity status—categorized as physically inactive or physically active. Physical activity status was assessed using WHO guidelines for 18 years and above^38^. Older adults who performed at least 75 min of vigorous-intensity physical activity or at least 150 min of moderate-intensity physical activity in a week or both were classified as “Physically active else, they were classified as “Physically inactive”.
- Difficulty in Activities of Daily Living (ADL) -categorized as no difficulty or faces difficulty. Information was collected on whether a person had difficulty dressing, walking across a room, bathing, eating, getting in or out of bed, and using the toilet. During the interview, older adults who faced difficulty in any one of the activities for more than three months were categorized as “faces difficulty.” Those with no difficulty in any activities were included in the “no difficulty” category^39^.
- Difficulty in Instrumental Activities of Daily Living -categorized as no difficulty or faces difficulty. LASI obtained information on whether an individual had difficulty in the following seven instrumental activities of daily living: preparing a hot meal, shopping for groceries, making telephone calls, taking medications, doing work around the house or garden, managing household finances and getting around or reaching an unfamiliar place. Those who faced difficulty in any one of the activities for more than three months during the interview were categorized as “faces difficulty” else were included in the “no difficulty” category^39^.
- Social participation—categorized as socially active or socially inactive). LASI collected information on whether a person goes out of the house for eating, goes outdoors for relaxing, plays indoor games, plays outdoor games or exercises, visits relatives, attends cultural events, attends religious functions, attends group meetings, reads books, newspaper and/or magazines, watches television or listens to the radio, use a computer for electronic communication. Older adults who has partaken in any of the above social activities were classified as “socially active” else were classified as “socially inactive”^40^.
- Satisfaction with the current living arrangement—categorized as satisfied, neutral, or not satisfied.
- Receipt of ill-treatment within past one year (Have you felt that you were ill-treated in the past year?) was- categorized as no or yes.

The socio-demographic characteristics of older adults included -

- Age (in years)
- Level of education (in years of schooling)
- Marital status categorized as currently married, currently not married, or widowed.
- Working status categorized as currently working, currently not working, never worked, or retired.

Household-related characteristics included -

- Monthly per capita consumption expenditure (MPCE) quintile of household categorized as poorest, poorer, middle, richer, richest.
- Major religions in India -categorized as Hinduism, Islam, or Other-.
- Caste -categorized as Scheduled Tribe (ST), Scheduled Caste (SC), Other Backward Class (OBC), or Other. The people in the ST and SC category belong to the most socially disadvantaged group, historically belonging to the lower rung of the now constitutionally abolished Indian caste system. People in the OBC category also belong to a socially and economically disadvantaged population group with better conditions than the SC/ST people. The “Others” category consisted of all people who did not belong to any of the three caste groups.
- Place of residence—categorized as urban or rural.

### Statistical methods

First, we computed older adults’ absolute and weighted percent distribution by background characteristics. Second, we assessed the men-women difference in major depression for the whole and the age-group-wise population using the proportion test. Third, using the bivariate analysis, we examined the unadjusted relationship between major depression and health, socio-demographic and household covariates using the chi-square test for association. Fourth, we assessed the association between major depression and the explanatory variables by multivariate logistic regression models. The odds ratios in our multivariable models provide the odds of depression relative to having no depression among older adults after adjusting for other explanatory variables^41^. Fifth, we conducted the above analysis separately for older men and older women.. None of our multivariate models violated the assumptions of multicollinearity^42^. Sixth, we utilized the nonlinear multivariate decomposition technique to identify the contribution of the explanatory factors to the men-women gap in major depression among older adults^43,44^. This decomposition analysis approached similarly to the mediation analysis to quantify the percentage of the total effect mediated by each factor in the model. We showed an overall and detailed decomposition of the gender difference in depression. In the overall decomposition, the gender difference in major depression was decomposed into endowments or characteristics (E) components and coefficients or an effects (C) component. The detailed decomposition provided the contribution of each health, socio-demographic and household-related explanatory characteristic to each endowment and coefficient component of the gender difference in depression. The endowments component (E) shows the expected change in the gender gap of depression if the distribution of men and women in older adults were similar for a particular category of the explanatory variable. Equivalently, the coefficients component (C) gives the expected change in the gender differential if older men and older women—had a similar risk of being in a particular category of the explanatory variable. Decomposition models can provide different estimates for different reference categories of the categorical covariates. We provide normalized decomposition estimates to overcome this “identification problem”^43,45^.

Lastly, our analysis was weighted to account for the—sample survey design of LASI. Details on the derivation of survey weights and their components are publicly available elsewhere^26^. We performed- all statistical estimations using the Stata Statistical Software, Version 15. https://www.stata.com/stata15/.

### Ethics approval and consent to participate

We used publicly available data with no information that could lead to the identification of the respondents. The Indian Council of Medical Research (ICMR) extended the necessary guidance and ethical approval for conducting the LASI. During the survey, LASI interviewers obtained informed consent from the respondents before data collection. The survey was implemented following the relevant guidelines and regulations.

## Results

### Descriptive statistics and bivariate analysis

**Figure 1:**
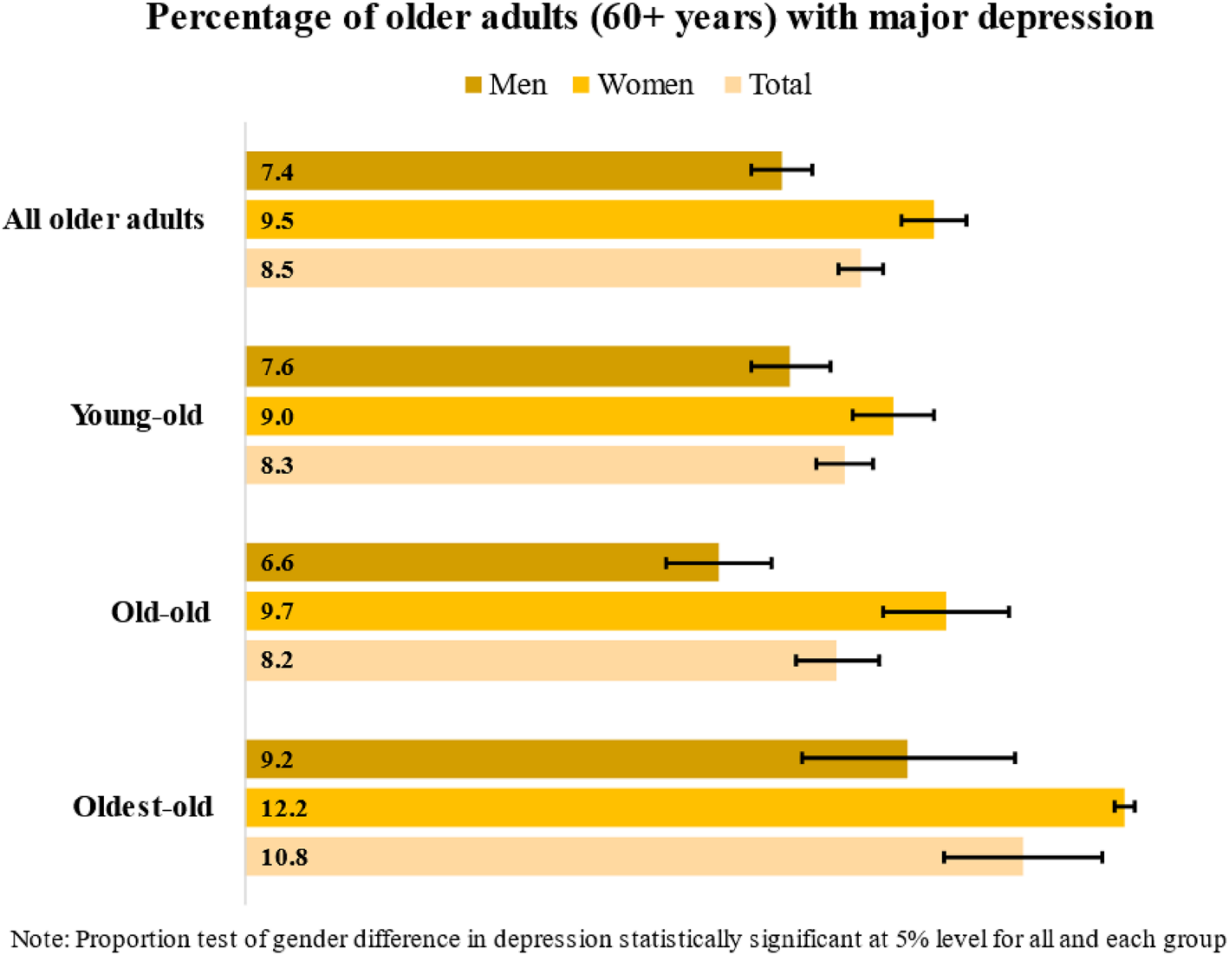
Proportion of men and women older adults with major depression in India, the Longitudinal Aging Study in India, 2017–2019.

Figure 1 depicts -proportion of self-reported depression among older men and women in India in 2017–2018. The overall depression—was higher among older women (9.5%, 95% CI 9.1, 10.0) [(95%, CI 9.1, 10.0) than men (7.4%, 95% CI 7.0, 7.8). Table 1 summarizes the bivariate association between depression and health-related, socio-demographic, and household characteristics of older men vs. older women in India in 2017–2018. Over one-fifth and one-fourth of older men and women reported poor self-rated health and multiple chronic conditions. A higher proportion of older men reported being physically active than older women (30% vs. 26%). However, greater percentage of older women reported ADL and IADL difficulty than men (ADL: 25% vs 20%; IADL, 56% vs. 38%). A higher proportion of older women were widowed, never worked, were socially inactive, received ill treatment from members within or outside the family and were not satisfied with their present living arrangements than men (Widow, 54% vs. 16%; never worked; 47% vs. 4%; socially inactive; 10% vs. 7%; ill-treatment, 6% vs. 5%).

**Table 1:** Distribution of sample characteristics and the bivariate association between depression and health-related, socio-demographic and household characteristics of older adults in India, the Longitudinal Aging Study in India, 2017–2019.

	Men older adults (60+ years)				Women older adults (60 + years)			
	Total	Depressed			Total	Depressed		
	N (WC %)	N	WR %	Chi2 (p-value)	N (WC %)	N	WR %	Chi2 (p-value)
Health-related characteristics								
Self-rated health								
Good	2374 (15.6)	57	3.9	346.35 (< 0.001)	1950 (11.8)	65	3.7	330.86 (< 0.001)
Average	9417 (63.3)	454	5.5		10,163 (63.3)	579	7.1	
Poor	2891 (21.1)	385	15.4		3842 (24.8)	536	17.6	
Chronic morbidity status								
No condition	7096 (49.5)	316	5.6	77.81 (< 0.001)	6955 (44.7)	433	8.3	46.65 (< 0.001)
Single condition	4170 (28.4)	282	7.5		4856 (30.0)	345	9.0	
Multiple conditions	3416 (22.1)	298	11.0		4144 (25.3)	402	11.5	
Physically active status	4056 (29.6)	240	6.7	0.33 (0.562)	3962 (25.9)	312	10.1	1.76 (0.184)
Faces difficulty in ADL	2534 (20.1)	315	14.3	214.03 (< 0.001)	3739 (25.4)	484	15.5	219.53 (< 0.001)
Faces difficulty in IADL	5035 (38.0)	500	10.9	195.93 (< 0.001)	8281 (56.3)	833	12.2	178.32 (< 0.001)
Socio-demographic characteristics								
Socially inactive	983 (7.4)	94	11.8	22.01 (< 0.001)	1436 (9.7)	166	12.8	39.95 (< 0.001)
Living arrangement satisfaction								
Satisfied	11,766 (77.6)	555	5.6	273.48 (< 0.001)	12,230 (72.5)	724	7.6	320.08 (< 0.001)
Neutral	2305 (17.6)	224	11.4		2901 (21.1)	273	9.9	
Not satisfied	611 (4.8)	117	20.2		824 (6.4)	183	26.7	
Received ill-treatment	537 (4.8)	107	19.9	185.85 (< 0.001)	733 (5.5)	165	23.8	256.26 (< 0.001)
Marital status								
Currently married	12,096 (81.1)	691	7.0	19.43 (< 0.001)	7420 (44.6)	472	8.4	28.53 (< 0.001)
Currently not married	388 (2.4)	26	5.7		403 (1.9)	20	7.8	
Widowed	2198 (16.5)	179	9.1		8132 (53.6)	688	10.1	
Working status								
Currently working	5963 (43.0)	312	6.1	30.50 (< 0.001)	2926 (19.2)	215	10.7	26.65 (< 0.001)
Currently not working	5740 (39.8)	427	8.6		4841 (32.3)	433	11.5	
Never worked	722 (3.8)	44	9.0		7818 (46.6)	509	7.2	
Retired	2257 (13.4)	113	6.9		370 (1.9)	23	12.0	
Household characteristics								
Household MPCE quintile								
Poorest	2928 (20.9)	173	7.3	10.67 (0.030)	3351 (22.5)	274	9.9	19.76 (0.001)
Poorer	2991 (21.7)	162	5.7		3313 (21.7)	248	9.2	
Middle	2978 (21.0)	170	7.6		3277 (20.5)	186	7.9	
Richer	2921 (19.4)	182	7.4		3109 (19.3)	235	9.4	
Richest	2864 (17.1)	209	9.0		2905 (16.0)	237	10.3	
Religion of household								
Hinduism	10,785 (82.8)	709	7.4	28.91 (< 0.001)	11,674 (82.6)	900	9.1	24.84 (< 0.001)
Islam	1751 (10.9)	111	7.3		1871 (10.6)	159	11.4	
Others	2146 (6.3)	76	5.9		2410 (6.8)	121	8.6	
Caste of household								
Scheduled Tribe	2371 (7.7)	71	4.0	56.84 (< 0.001)	2654 (8.4)	93	5.4	79.41 (< 0.001)
Scheduled Caste	2380 (19.0)	175	9.6		2625 (19.0)	239	10.0	
Other Backward Class	5634 (45.6)	400	7.3		5960 (44.9)	503	10.3	
Others	4297 (27.7)	250	6.7		4716 (27.7)	345	8.5	
Rural place of residence	9816 (72.7)	647	7.8	12.33 (< 0.001)	10,390 (69.3)	869	10.7	40.75 (< 0.001)
Overall	14,682	896	7.3		15,955	1,180	9.3	

(a) N: Sample size, WC_%: Weighted column percentage, WR_%: Weighted row percentage, Chi^2^: Chi-square test statistic; (b) ADL: Activities of daily living, IADL: Instrumental activities of daily living, MPCE: Monthly per capita expenditure.

### Factors associated with higher prevalence of depression in older adults

Factors associated with a higher percentage of depression in older men vs. older women included the following: poor self-rated health (15.4% vs. 17.6%), multiple chronic conditions (11.0% vs. 11.5%), difficulty in ADL (14.3% vs. 15.5%;), difficulty in IADL (10.9% vs. 12.2%), being socially inactive (11.8% vs. 12.8), lack of satisfaction with present living arrangement (20.2% vs 26.7%), being ill-treated (19.9% vs. 23.8%), being widowed (9.1% vs. 10.1), and having a rural residence (7.8% vs. 10.7%).

Supplementary Table S1 shows the distribution of older adults in the complete and current datasets, including missing values. The sensitivity check shows the distribution of older adults by independent characteristics is similar between the complete and current datasets.

### Multivariable analysis

Table 2 summarizes the results of multivariable logistic regression. Poor self-rated health, multimorbidity, physical activity, and difficulty in ADL and IADL were health-related factors with higher odds of depression among older men and older women. Being socially inactive, not being satisfied with once-present living arrangements, receiving ill-treatment, and being widowed were socio-demographic factors with higher odds of depression among older men and older women. Additionally, belonging to SC and having rural residence also had higher odds of depression among older men and older women in India.

**Table 2:** Multivariable logistic regression analysis of depression and health-related, socio-demographic and household characteristics of older adults in India, the Longitudinal Aging Study in India, 2017–2019.

	Depression among older adults (60+ years)			
	Men		Women	
	OR	95% CI	OR	95% CI
Health-related characteristics				
Self-rated health [ref = (Good)]				
Average	1.50***	(1.14, 1.98)	1.25*	(0.97, 1.62)
Poor	2.95***	(2.19, 3.95)	2.28***	(1.74, 2.98)
Chronic morbidity status (ref = No condition)				
Single condition	1.31***	(1.10, 1.56)	1.05	(0.90, 1.22)
Multiple conditions	1.40***	(1.16, 1.68)	1.32***	(1.13, 1.55)
Physical activity status (ref = Physically inactive)				
Physically active	1.23**	(1.03, 1.48)	1.31***	(1.12, 1.52)
Difficulty in ADL (ref = No difficulty)				
Faces difficulty	1.63***	(1.37, 1.93)	1.67***	(1.45, 1.92)
Difficulty in IADL (ref = No difficulty)				
Faces difficulty	1.57***	(1.34, 1.86)	1.42***	(1.23, 1.65)
Socio-demographic characteristics				
Social participation (ref = Socially active)				
Socially inactive	1.33**	(1.04, 1.70)	1.38***	(1.15, 1.67)
Living arrangement satisfaction (ref = Satisfied)				
Neutral	1.68***	(1.42, 2.00)	1.27***	(1.09, 1.48)
Not satisfied	2.69***	(2.12, 3.42)	2.45***	(2.01, 2.98)
Received ill-treatment (ref = No)				
Yes	2.79***	(2.20, 3.54)	2.56***	(2.10, 3.13)
Age (in years)	0.97***	(0.96, 0.98)	0.99	(0.99, 1.00)
Level of education (in years)	1.00	(0.98, 1.02)	0.99	(0.97, 1.01)
Marital status (ref = Currently married)				
Currently not married	0.92	(0.60, 1.41)	0.69	(0.43, 1.11)
Widowed	1.49***	(1.24, 1.79)	1.20***	(1.05, 1.38)
Working status [ref = (Currently working)]				
Currently not working	1.10	(0.92, 1.32)	0.94	(0.78, 1.13)
Never worked	1.06	(0.74, 1.50)	0.78***	(0.64, 0.94)
Retired	1.03	(0.80, 1.34)	0.94	(0.58, 1.51)
Household characteristics				
Household MPCE quintile (ref = Poorest)				
Poorer	0.96	(0.77, 1.21)	0.97	(0.80, 1.16)
Middle	1.05	(0.84, 1.32)	0.75***	(0.61, 0.91)
Richer	1.27**	(1.02, 1.60)	0.98	(0.81, 1.19)
Richest	1.46***	(1.16, 1.83)	1.11	(0.91, 1.35)
Religion of household (ref = Hinduism)				
Islam	0.99	(0.80, 1.24)	1.12	(0.93, 1.36)
Others	0.72**	(0.56, 0.93)	0.99	(0.80, 1.22)
Caste of household (ref = Scheduled tribe)				
Scheduled caste	1.70***	(1.26, 2.28)	2.24***	(1.73, 2.91)
Other backward class	1.77***	(1.34, 2.32)	2.28***	(1.79, 2.92)
Others	1.58***	(1.18, 2.10)	2.22***	(1.72, 2.87)
Place of residence (ref = Urban)				
Rural	1.16*	(0.98, 1.38)	1.44***	(1.24, 1.67)
Analytical sample size	14,682		15,955	

(a) OR: Odds ratio, CI: Confidence Interval, ref: Reference category; (b) Statistical significance denoted by asterisks where * p-value < 0.1, ** p-value < 0.05 and *** p-value < 0.01; (c) ADL: Activities of daily living, IADL: Instrumental activities of daily living, MPCE: Monthly per capita expenditure.

### Decomposing gender gap in depression

Table 3 describes the overall decomposition of the men-women gap in depression among older adults in India for 2017–2018. We found differences in effects (C) accounted for 19.2% of the observed gender differences in the proportion of depression. However; 80.8% of the gender differentials in depression were explained by differences in compositional characteristics (E); which is the counterfactual comparison of the difference in outcomes from men’s group i.e., the expected difference if men were given women’s group distribution of covariates. Similarly, C is the counterfactual comparison of outcomes from women’s group perspective i.e., the expected difference if women experienced men’s group behavioural responses to covariates.

**Table 3:** Overall decomposition of men-women gap in major depression among older adults in India, the Longitudinal Aging Study in India, 2017–2019.

Component	Men-Women gap in depression among older adults			
	Coefficient	95% CI	*p* value	Percent
Explained difference (E)	0.01109	(0.00521, 0.01697)	< 0.001	80.8
Unexplained difference (C)	0.00264	(− 0.00514, 0.01042)	0.506	19.2
Raw difference (R)	0.01373	(0.00825, 0.01920)	< 0.001	100.0

CI Confidence Interval.

Table 4 summarizes decomposition of the men-women gap in depression among older adults. We provide differences due to characteristics and find marital status contributes to 36.7% of the gender gap in depression among older adults. Further, ADL and IADL difficulties among men and women contributed to gender gap by 17.6% and 34.0%, respectively. Additionally, self-rated health contributed to men-women gap by 18.8%, whereas, having social participation and satisfaction in present living arrangements contributed to 4.4% and 8.1% -gender gap in depression.

**Table 4:** Detailed decomposition (with normalized coefficients) of men–women gap in depression among older adults in India, the Longitudinal Aging Study in India, 2017–2019.

	Men–women gap in depression among older adults (60+ years)							
	Difference due to characteristics (E)				Difference due to coefficients (C)			
	Coefficient	95% CI	*p* value	Percent	Coefficient	95% CI	*p* value	Percent
Health-related characteristics								
Self-rated health								
Good	0.00101	(0.00049, 0.00153)	< 0.001	7.4	0.00058	(− 0.00072, 0.00187)	0.383	4.2
Average	0.00004	(0.00000, 0.00008)	0.028	0.3	− 0.00057	(− 0.00316, 0.00202)	0.667	− 4.1
Poor	0.00152	(0.00102, 0.00202)	< 0.001	11.1	− 0.00053	(− 0.00167, 0.00060)	0.359	− 3.9
Chronic morbidity status								
No condition	0.00038	(0.00006, 0.00071)	0.022	2.8	0.00113	(− 0.00131, 0.00356)	0.365	8.2
Single condition	− 0.00009	(− 0.00023, 0.00004)	0.167	− 0.7	− 0.00093	(− 0.00273, 0.00088)	0.314	− 6.7
Multiple conditions	0.00035	(0.00015, 0.00056)	0.001	2.6	0.00022	(− 0.00069, 0.00113)	0.638	1.6
Physical activity status								
Physically inactive	− 0.00026	(− 0.00042, − 0.00011)	0.001	− 1.9	− 0.00050	(− 0.00276, 0.00176)	0.665	− 3.6
Physically active	− 0.00026	(− 0.00042, − 0.00011)	0.001	− 1.9	0.00018	(− 0.00065, 0.00102)	0.665	1.3
Difficulty in ADL								
No difficulty	0.00121	(0.00077, 0.00164)	< 0.001	8.8	− 0.00027	(− 0.00260, 0.00206)	0.819	− 2.0
Faces difficulty	0.00121	(0.00077, 0.00164)	< 0.001	8.8	0.00006	(− 0.00045, 0.00057)	0.819	0.4
Difficulty in IADL								
No difficulty	0.00233	(0.00127, 0.00339)	< 0.001	17.0	0.00081	(− 0.00138, 0.00300)	0.470	5.9
Faces difficulty	0.00233	(0.00127, 0.00339)	< 0.001	17.0	− 0.00043	(− 0.00161, 0.00074)	0.470	− 3.2
Socio-demographic characteristics								
Social participation								
Socially active	0.00030	(0.00011, 0.00049)	0.002	2.2	− 0.00045	(− 0.00411, 0.00320)	0.808	− 3.3
Socially inactive	0.00030	(0.00011, 0.00049)	0.002	2.2	0.00004	(− 0.00025, 0.00032)	0.808	0.3
Living arrangement satisfaction								
Satisfied	0.00099	(0.00065, 0.00133)	< 0.001	7.2	0.00249	(− 0.00238, 0.00736)	0.316	18.1
Neutral	− 0.00026	(− 0.00046, − 0.00005)	0.015	− 1.9	− 0.00059	(− 0.00173, 0.00056)	0.314	− 4.3
Not satisfied	0.00039	(0.00025, 0.00052)	0.000	2.8	0.00003	(− 0.00018, 0.00024)	0.773	0.2
Received ill-treatment								
No	0.00033	(0.00022, 0.00043)	< 0.001	2.4	0.00101	(− 0.00302, 0.00505)	0.623	7.4
Yes	0.00033	(0.00022, 0.00043)	< 0.001	2.4	− 0.00004	(− 0.00019, 0.00011)	0.623	− 0.3
Age (in years)	0.00013	(− 0.00006, 0.00031)	0.185	0.9	0.04548	(− 0.03642, 0.12738)	0.276	331.3
Level of education (in years)	0.00210	(− 0.00281, 0.00702)	0.401	15.3	− 0.00145	(− 0.00523, 0.00232)	0.451	− 10.6
Marital status								
Currently married	− 0.00167	(− 0.00640, 0.00307)	0.491	− 12.1	0.00341	(− 0.00443, 0.01125)	0.394	24.8
Currently not married	0.00003	(0.00000, 0.00007)	0.066	0.2	− 0.00008	(− 0.00040, 0.00024)	0.618	− 0.6
Widowed	0.00668	(0.00181, 0.01154)	0.007	48.6	− 0.00018	(− 0.00117, 0.00081)	0.725	− 1.3
Working status								
Currently working	− 0.00158	(− 0.00438, 0.00121)	0.268	− 11.5	0.00142	(− 0.00194, 0.00477)	0.409	10.3
Currently not working	− 0.00021	(− 0.00117, 0.00074)	0.664	− 1.5	− 0.00020	(− 0.00215, 0.00176)	0.844	− 1.4
Never worked	− 0.00521	(− 0.01075, 0.00033)	0.065	− 38.0	− 0.00021	(− 0.00075, 0.00033)	0.454	− 1.5
Retired	− 0.00030	(− 0.00363, 0.00304)	0.861	− 2.2	0.00016	(− 0.00136, 0.00168)	0.833	1.2
Household characteristics								
Household MPCE quintile								
Poorest	0.00003	(− 0.00005, 0.00012)	0.446	0.3	0.00087	(− 0.00088, 0.00261)	0.330	6.3
Poorer	0.00000	(− 0.00004, 0.00005)	0.842	0.0	0.00089	(− 0.00087, 0.00266)	0.322	6.5
Middle	− 0.00003	(− 0.00005, − 0.00001)	0.001	− 0.2	− 0.00084	(− 0.00258, 0.00090)	0.343	− 6.1
Richer	− 0.00001	(− 0.00004, 0.00003)	0.621	− 0.1	− 0.00041	(− 0.00154, 0.00072)	0.474	− 3.0
Richest	− 0.00015	(− 0.00027, − 0.00002)	0.021	− 1.1	− 0.00048	(− 0.00169, 0.00072)	0.432	− 3.5
Religion of household								
Hinduism	0.00001	(− 0.00002, 0.00003)	0.511	0.1	− 0.00266	(− 0.00796, 0.00264)	0.325	− 19.4
Islam	− 0.00001	(− 0.00003, 0.00001)	0.256	− 0.1	− 0.00007	(− 0.00069, 0.00056)	0.827	− 0.5
Others	− 0.00002	(− 0.00007, 0.00004)	0.528	− 0.1	0.00062	(− 0.00066, 0.00190)	0.343	4.5
Caste of household								
Scheduled Tribe	− 0.00027	(− 0.00037, − 0.00017)	< 0.001	− 2.0	− 0.00088	(− 0.00280, 0.00104)	0.368	− 6.4
Scheduled Caste	0.00004	(0.00001, 0.00006)	0.004	0.3	0.00024	(− 0.00066, 0.00113)	0.602	1.7
Other Backward Class	− 0.00016	(− 0.00025, − 0.00008)	< 0.001	− 1.2	0.00036	(− 0.00126, 0.00198)	0.664	2.6
Others	0.00002	(0.00001, 0.00004)	0.003	0.2	0.00090	(− 0.00112, 0.00291)	0.383	6.5
Place of residence								
Urban	− 0.00023	(− 0.00034, − 0.00011)	< 0.001	− 1.7	− 0.00087	(− 0.00269, 0.00094)	0.346	− 6.4
Rural	− 0.00023	(− 0.00034, − 0.00011)	< 0.001	− 1.7	0.00176	(− 0.00189, 0.00541)	0.346	12.8
Constant	–	–	–	–	− 0.04735	(− 0.13127, 0.03657)	0.269	− 345.0

(a) CI: Confidence interval; (b) ADL: Activities of daily living, IADL: Instrumental activities of daily living, MPCE: Monthly per capita expenditure.

## Discussion

Our study aimed to find the factors associated with the percentage of self-reported depression among older men and older women and to identify factors contributing to the gender difference in the percentage of depression in India. Prior research has demonstrated a higher prevalence of depression among women globally^46–50^ with the rates of depression being—nearly two-fold higher among women than men due to psychosocial mediators such as obesity and perceived interpersonal and behavioural issues^47,51,52^.

Our findings suggest -chronic diseases, disability and higher body mass index contribute to the gender differentials in depression. Consistently, studies have found a strong relationship between body mass index which is highly correlated with physical health status and depression among women^49^. Similarly, other studies found factors such as poor self-perceived health, physical health, physical inactivity, life-limiting and long-term illness or pain, higher level of functional impairment, and decline in cognitive capacity to be associated with an increased likelihood of depression among older women^22,30,50,53,54^. These findings underscore the need for developing gender-based interventions to minimize the negative impact of physical health problems and obesity and obesity-associated multimorbidity on mental health of older women. Additionally, prior studies suggest being physically active and exercising could be important non-pharmacological interventions for lowering both anxiety and depression among older women^49^.

An interesting finding of our study was social participation, working status and place of residence were found to be significant factors associated with higher percentage of depression in women. Our result is similar to a report by World Health Organization on mental health which states the key social factors for improving mental health in women are predominantly social inclusion, provision of adequate income, and social supports and services^55^. Another study showed a decline in the relative well-being of women among those who remained at home, those who were under pressure to be “successful” at work, as a family member or as an important member and contributor to the society^56^. Similarly, marital transitions such as separation/divorce and widowhood are major risk factors for depression among women as these life events may signal loneliness onset. It is noteworthy to highlight older women have higher -chance of -living in a solo arrangement. Additionally, social capital through civic participation may have differential effects on men and women^57^. Furthermore, low spousal support and extended family relationship strains have been strongly associated with mental illnesses among older women. Women are also less likely to have greater social networks which are protective factors for—loneliness and depression^58,59^. An interestingly finding of our study is a stronger association of depression in widowed men than in widowed women. This finding can explain the increased stress among men than women due to spousal death and the resultant lack of care and social and mental support and cohesion that is foregone on familial and societal level for which women are the primary conduits in a family^35,60,61^.

In the -socio-cultural setting of India, life cycle vulnerabilities, gender-based violence, limited educational opportunities, economic distress, limited autonomy, and minimal family support are a few prominent factors that increase-depressive symptoms specifically among women^7,16,18–20,62^. This is -similar to by prior research that reported increased contribution of household characteristics, such as household wealth status, religious and social group status and rural/urban residential status, as risk factors to the increased prevalence of depression among older women in comparison to older men. Additionally, other prior findings have showed rural place of residence and migration of adult male children to cities for job or better life can increase the likelihood of depressive symptoms among older adults and among older women or mothers in particular^63,64^. All these and our findings combined suggests the urgent and critical need for gender-specific policy formulation to address- the socioeconomic disadvantages and increased burden of mental illnesses among older women in India.

A number of other reasons may also explain why the prevalence of depression is higher among women than men. Previous studies have suggested differences in help seeking behaviour, gender roles, and social and biological factors^65–67^ which may contribute to depression among older adults. Women may have different age of onset, disease course, internalizing factors for depression, symptom profile of mental illnesses and may be more willing to admit and report the symptoms and affective depressed feelings when asked in comparison to men^68,69^. Financial or economic dependencies on others/spouse is another major factor that can lead to increased risk of late-life mental illnesses and depression specifically among older women^35,70^. Parker and Brotchie explain women are either differentially exposed to a greater number of life stressors and/or are more vulnerable to them^66^. Additionally, over time, men are more likely to forget episodes (or remember fewer symptoms) while women are more likely to remember them which can lead to reporting biases^56^. Men and women also have different coping mechanisms as men are more likely to enhance their wellbeing by increasing their sports activity, alcohol consumption, participation in civic or community engagement activities whereas women cope through emotional release or by internalizing them which can negatively impact their health and/or exacerbate their existing disease profile^71^. These need to be further investigated in particular regional settings, villages and rural settings of India.

### Limitations and strength

We acknowledge several limitations.. First, due to cross-sectional design of our study, it is difficult to resolve the causal direction of the observed associations. That is, we cannot -tease out the -causal association between physical health and chronic conditions due to their bi-directional association with depression. Second, -reverse causality in some of the observed associations is -possible. We cannot rule out poor mental health can lead to decline in social networks and social participation. Therefore, studies with longitudinal and prospective cohort designs in which the cause-and-effect pathway is more reliable are necessary for identifying causal relationships between the predictor variables and depression. Third, it is possible some of the correlates in our multivariate models are likely in the pathway of other correlates (e.g., indicators such as social participation or physical activity could likely be in the pathway of other health-related indicators; As such including these confounding variables in the same models simultaneously may artificially reduce the variance “explained” by the preceding indicators, particularly when coefficients are interpreted as a standalone. Our study has several strengths. Our sample provides comprehensive information on socioeconomics and physical and mental health of older adults from a large nationally representative survey which provides external validity to all the States and Union Territories of India thereby expanding the generalizability of our findings. The results may be also applicable to other LMIC’s and could aid in cross-country comparative studies on global depression among older adults both from high income and LMIC’s around the globe.

## Conclusions

Our study analyzed factors that explain the difference in the percentages of depression among older men and older women in India. We found various socioeconomic, demographic, and health-related factors that explain the difference in depression proportion between men vs. women. The most significant factors associated with depression were age, poor self-rated health, multiple chronic conditions, functional limitation, living arrangement and marital status. Other factors such as social participation, working status, and place of residence were found to be significant for women only. Our study highlights that depression is an emerging major public health problem with a greater female disadvantage that needs critical and culturally appropriate policy solutions for combating depression, social isolation and loneliness among older adults. These policy solutions could involve (1) re-designing co-residential homes for multiple generations of family to live together all in one building such as in Singapore which now is declared as a man-made blue zone, (2) creation of loneliness ministry as in Japan or the (3) development of high quality, socially connected and culturally tailored old age homes for those who prefer to age in their homes or in their communities to age and thrive. In conclusion, our findings provide a broader framework for policy makers and health practitioners to focus on gender and older adult specific policies to mitigate depression among older adults and among older women in in India.

### Supplementary Information

Supplementary Table 1.

## Data Availability

Our study utilizes a secondary data that is freely available in the public domain through the International Institute of Population Sciences data request form available at https://iipsindia.ac.in/sites/default/files/LASI_DataRequestForm_0.pdf and from the Gateway to Global Aging from https://g2aging.org/.
