# Supplementary material for: Depression by gender and associated factors among older adults in India: implications for age-friendly policies
Source: Sci Rep. 2023 Oct 17;13:17651. doi: 10.1038/s41598-023-44762-8 (PMC10582097; doi:10.1038/s41598-023-44762-8)
Supplement: Supplementary file 1 — Supplementary Table 1. [file 41598_2023_44762_MOESM1_ESM.docx]

**Supplementary Files**

**Table S1: Absolute and percentage distribution of older adults in the complete and current (with missing values) datasets**

| **Characteristics** | **Complete dataset** | |  | **Current dataset** | |  | **Abs Diff** |
| --- | --- | --- | --- | --- | --- | --- | --- |
|  | **N** | **%** |  | **N** | **%** |  | **%** |
| ***Health-related characteristics*** |  |  |  |  |  |  |  |
| **Self-rated health** |  |  |  |  |  |  |  |
| Good | 4,349 | 13.8 |  | 4,324 | 14.1 |  | 0.3 |
| Average | 20,342 | 64.7 |  | 19,580 | 63.9 |  | 0.8 |
| Poor | 6,773 | 21.5 |  | 6,733 | 22.0 |  | 0.5 |
| **Chronic morbidity status** |  |  |  |  |  |  |  |
| No condition | 14,420 | 45.8 |  | 14,051 | 45.9 |  | 0.1 |
| Single condition | 9,245 | 29.4 |  | 9,026 | 29.5 |  | 0.1 |
| Multiple conditions | 7,799 | 24.8 |  | 7,560 | 24.7 |  | 0.1 |
| **Physical activity status** |  |  |  |  |  |  |  |
| Physically inactive | 23,408 | 74.4 |  | 22,619 | 73.8 |  | 0.6 |
| Physically active | 8,056 | 25.6 |  | 8,018 | 26.2 |  | 0.6 |
| **Difficulty in ADL** |  |  |  |  |  |  |  |
| No difficulty | 24,770 | 78.7 |  | 24,364 | 79.5 |  | 0.8 |
| Faces difficulty | 6,694 | 21.3 |  | 6,273 | 20.5 |  | 0.8 |
| **Difficulty in IADL** |  |  |  |  |  |  |  |
| No difficulty | 17,609 | 56.0 |  | 17,321 | 56.5 |  | 0.5 |
| Faces difficulty | 13,855 | 44.0 |  | 13,316 | 43.5 |  | 0.5 |
| ***Socio-demographic characteristics*** |  |  |  |  |  |  |  |
| **Level of life satisfaction** |  |  |  |  |  |  |  |
| Satisfied | 15,065 | 47.9 |  | 14,933 | 48.7 |  | 0.8 |
| Somewhat satisfied | 12,895 | 41.0 |  | 12,231 | 39.9 |  | 1.1 |
| Not satisfied | 3,504 | 11.1 |  | 3,473 | 11.3 |  | 0.2 |
| **Social participation** |  |  |  |  |  |  |  |
| Socially active | 28,782 | 91.5 |  | 28,218 | 92.1 |  | 0.6 |
| Socially inactive | 2,682 | 8.5 |  | 2,419 | 7.9 |  | 0.6 |
| **Living arrangement satisfaction** |  |  |  |  |  |  |  |
| Satisfied | 24,816 | 78.9 |  | 23,996 | 78.3 |  | 0.6 |
| Neutral | 5,212 | 16.6 |  | 5,206 | 17.0 |  | 0.4 |
| Not satisfied | 1,436 | 4.6 |  | 1,435 | 4.7 |  | 0.1 |
| **Received ill-treatment** |  |  |  |  |  |  |  |
| No | 30,194 | 96.0 |  | 29,367 | 95.9 |  | 0.1 |
| Yes | 1,270 | 4.0 |  | 1,270 | 4.1 |  | 0.1 |
| **Age group** |  |  |  |  |  |  |  |
| Oldest-old | 3,389 | 10.8 |  | 3,127 | 10.2 |  | 0.6 |
| Old-old | 9,101 | 28.9 |  | 8,843 | 28.9 |  | 0.0 |
| Young-old | 18,974 | 60.3 |  | 18,667 | 60.9 |  | 0.6 |
| **Level of education** |  |  |  |  |  |  |  |
| Secondary and above | 7,015 | 22.3 |  | 6,859 | 22.4 |  | 0.1 |
| Upto primary | 7,560 | 24.0 |  | 7,390 | 24.1 |  | 0.1 |
| No formal education | 16,889 | 53.7 |  | 16,388 | 53.5 |  | 0.2 |
| **Marital status** |  |  |  |  |  |  |  |
| Currently married | 19,920 | 63.3 |  | 19,516 | 63.7 |  | 0.4 |
| Currently not married | 825 | 2.6 |  | 791 | 2.6 |  | 0.0 |
| Widowed | 10,719 | 34.1 |  | 10,330 | 33.7 |  | 0.4 |
| **Working status** |  |  |  |  |  |  |  |
| Currently working | 8,997 | 28.6 |  | 8,889 | 29.0 |  | 0.4 |
| Currently not working | 10,990 | 34.9 |  | 10,581 | 34.5 |  | 0.4 |
| Never worked | 8,784 | 27.9 |  | 8,540 | 27.9 |  | 0.0 |
| Retired | 2,693 | 8.6 |  | 2,627 | 8.6 |  | 0.0 |
| ***Household characteristics*** |  |  |  |  |  |  |  |
| **Household MPCE quintile** |  |  |  |  |  |  |  |
| Poorest | 6,484 | 20.6 |  | 6,279 | 20.5 |  | 0.1 |
| Poorer | 6,477 | 20.6 |  | 6,304 | 20.6 |  | 0.0 |
| Middle | 6,416 | 20.4 |  | 6,255 | 20.4 |  | 0.0 |
| Richer | 6,170 | 19.6 |  | 6,030 | 19.7 |  | 0.1 |
| Richest | 5,917 | 18.8 |  | 5,769 | 18.8 |  | 0.0 |
| **Religion of household** |  |  |  |  |  |  |  |
| Hinduism | 23,037 | 73.2 |  | 22,459 | 73.3 |  | 0.1 |
| Islam | 3,731 | 11.9 |  | 3,622 | 11.8 |  | 0.1 |
| Others | 4,696 | 14.9 |  | 4,556 | 14.9 |  | 0.0 |
| **Caste of household** |  |  |  |  |  |  |  |
| Scheduled Tribe | 5,173 | 16.4 |  | 5,025 | 16.4 |  | 0.0 |
| Scheduled Caste | 5,140 | 16.3 |  | 5,005 | 16.3 |  | 0.0 |
| Other Backward Class | 11,886 | 37.8 |  | 11,594 | 37.8 |  | 0.0 |
| Others | 9,265 | 29.4 |  | 9,013 | 29.4 |  | 0.0 |
| **Place of residence** |  |  |  |  |  |  |  |
| Urban | 10,739 | 34.1 |  | 10,431 | 34.0 |  | 0.1 |
| Rural | 20,725 | 65.9 |  | 20,206 | 66.0 |  | 0.1 |
| **Country region** |  |  |  |  |  |  |  |
| Southern | 7,578 | 24.1 |  | 7,366 | 24.0 |  | 0.1 |
| Northern | 7,981 | 25.4 |  | 7,788 | 25.4 |  | 0.0 |
| Central | 2,093 | 6.7 |  | 2,046 | 6.7 |  | 0.0 |
| Western | 4,303 | 13.7 |  | 4,178 | 13.6 |  | 0.1 |
| Eastern | 5,757 | 18.3 |  | 5,619 | 18.3 |  | 0.0 |
| North-eastern | 3,752 | 11.9 |  | 3,640 | 11.9 |  | 0.0 |
| **Overall** | **31,464** | **100** |  | **30,637** | **100** |  | **0** |

**Note – (a) N: Sample size, %: Percentage, Abs Diff: Absolute difference in the percentage of older adults between the complete and current datasets.**
